# Self-Supporting Sn-Based Carbon Nanofiber Anodes for High-Performance Lithium-Ion Batteries

**DOI:** 10.3390/molecules30081740

**Published:** 2025-04-13

**Authors:** Jingjie Xie, Lan Xu

**Affiliations:** 1 National Engineering Laboratory for Modern Silk, College of Textile and Clothing Engineering, Soochow University, Suzhou 215123, China; xiejingjie2023@163.com; 2 Jiangsu Engineering Research Center of Textile Dyeing and Printing for Energy Conservation, Discharge Reduction and Cleaner Production (ERC), Soochow University, Suzhou 215123, China

**Keywords:** Sn-based materials, carbon nanofibers, electrospinning, self-supporting anode, lithium-ion batteries

## Abstract

Due to its high theoretical specific capacity, abundant resources, accessibility and environmental friendliness, Sn has been considered as a promising alternative to lithium-ion batteries (LIBs) anodes. However, Sn anodes still face great challenges such as huge volume change and low conductivity. Herein, a self-supporting Sn-based carbon nanofiber anode for high-performance LIBs was prepared. Sn-based nanoparticles with high theoretical specific capacity were uniformly embedded in carbon nanofibers, which not only mitigated the volume expansion of Sn-based nanoparticles, but also obtained composite carbon nanofibers with excellent mechanical properties by adjusting the ratio of polyacrylonitrile to polyvinylpyrrolidone, exhibiting excellent electrochemical performance. The obtained optimal self-supporting Sn-based carbon nanofiber anode (Sn-SnO_2_/CNF-2) showed a discharge specific capacity of 607.28 mAh/g after 100 cycles at a current density of 500 mA/g. Even after 200 cycles, Sn-SnO_2_/CNF-2 still maintained a capacity of 543.78 mAh/g and maintained its original fiber structure well, demonstrating its good long-term cycling stability. This indicated that the self-supporting Sn-SnO_2_/CNF-2 anode had great potential for advanced energy storage.

## 1. Introduction

Lithium-ion batteries (LIBs) are increasingly being used because of their high energy density, good cycling performance, rechargeability, and light weight. They play a very important role in many fields such as mobile phones, electric vehicles, and aerospace, bringing great convenience [1,2,3]. Among them, the anode materials have a very important influence on the performance of LIBs. Currently, graphite is commonly used as an anode material for LIBs, but its low specific capacity (about 372 mAh/g) results in poor electrochemical performance of the prepared LIBs [4,5,6]. In order to meet the rapidly growing demand for batteries in multiple fields, it is necessary to develop new anode materials with higher energy and power density [7]. Sn has received widespread attention due to its high theoretical specific capacity (about 994 mAh/g) and relatively low average delithiation potential (~0.6 V), as well as its abundant content in nature, low price, and environmentally friendly characteristics [8,9]. However, its volume expansion during charging and discharging can be as high as 260%, which leads to structural collapse and the formation of an unstable solid electrolyte interface (SEI), severely weakening the lithium storage capacity and cycling performance of LIBs [10,11]. Therefore, the rapid decline in battery capacity caused by the large volume change in Sn anode materials poses a challenge to their practical application in LIBs.

In order to solve the above problems of Sn anode materials, they are often combined with carriers such as carbon nanofibers to improve their electronic/ionic conductivity and buffer the structural collapse induced by their volume changes [12]. For example, Li et al. [13] prepared Sn-SnSb/PCNFs composites consisting of porous carbon nanofibers and Sn-SnSb nanoparticles by electrospinning and carbonization processes, and the discharge specific capacity was still up to 447 mAh/g after 600 cycles at a current density of 1 A/g. In addition, Zhang et al. [14] designed SnO_2_/ZnO porous hollow nanotubes using polyvinylpyrrolidone (PVP) with different molecular weights by electrospinning, and prepared SnO_2_/ZnO@PPy anode by in situ polymerization of polypyrrole (PPy) on their surface. Yu et al. [15] synthesized SnP_2_O_7_/rGO composites by liquid nitrogen rapid freezing and subsequent annealing. The combination of SnP_2_O_7_ sheets and gauzy-like rGO significantly improved the kinetics of electrochemical reactions in the SnP_2_O_7_/rGO composite, resulting in its outstanding rate capability. Tout et al. [16] explored the effect of nanostructures on the electrochemical performance of SnHPO_3_ as anode materials, and its reversible capacity of 1008 mAh/g could be achieved at 0.2 C current density for the first cycle. Meanwhile, Idrissi et al. [17] studied the lithiation mechanism of SnHPO_3_ as LIB anodes, indicating that the amorphous phosphite adapted well to the volume changes in Sn particles during cycling, and the SnHPO_3_ electrode showed promising cycling stability. Currently, many studies on LIB anode materials involve preparing active materials into a slurry and coating it onto a metal foil to prepare conventional rigid anodes. It is often necessary to add binders and conductive agents for bonding in this preparation process, which can lead to a decrease in the adhesion between the slurry and the metal foil during repeated charging and discharging processes, and even cause the detachment of active materials, thereby reducing the utilization rate of active materials to a certain extent [18,19]. Therefore, the research on self-supporting anodes that do not require the addition of electrochemically inactive materials is particularly important.

Generally, there are two strategies for the preparation of self-supporting anodes [20]: (1) Using self-supporting conductive materials (carbon cloth, foam metal, sponge, etc.) as a skeleton, and loading active materials onto it to ensure that the two are tightly bonded; (2) Directly preparing electrochemically active materials into self-supporting anodes, usually using active materials such as carbon fibers, carbon nanotubes, graphene, and conductive polymers as substrates. Among them, carbon fibers, due to their unique network structure and good stability, play an important role as a key material for suppressing volume changes in flexible batteries during the lithiation–delithiation process. Therefore, using electrospun composite carbon nanofiber membranes (NFMs) directly as self-supporting anodes can maximize the utilization of active materials [21,22].

Self-supporting anodes require anode materials to have good mechanical properties, especially a certain degree of flexibility [23]. Polyacrylonitrile (PAN) is commonly used as the main carbon precursor for electrospun carbon nanofibers due to its high carbon yield and excellent thermal stability [24]. In addition, the linear configuration of PAN nanofibers can be transformed into an aromatic structure similar to graphite through high-temperature carbonization [25]. However, the carbon NFMs obtained from them are brittle and have poor flexibility, which limits their application in self-supporting anodes. PVP has abundant nitrogen-containing functional groups, and the doping of PVP can promote nitrogen doping during carbonization [26]. Meanwhile, it has also been shown that PVP has good flexibility [27]. Therefore, in this paper, Sn-based materials with high theoretical specific capacity were combined with PAN and PVP polymers as carbon precursors to prepare Sn-carbon (Sn-C) NFMs using electrospinning and high-temperature carbonization, which were directly used as self-supporting anodes for LIBs. By exploring different mass ratios of PAN and PVP, the optimal mass ratio was determined, which enabled the prepared optimal Sn-C NFM anode to have excellent mechanical properties and encapsulate the Sn-based nanoparticles well, effectively mitigating the problem of their volume change. Accordingly, the optimal self-supporting Sn-C NFM anode exhibited excellent electrochemical performance, indicating its promising application in flexible energy storage devices.

## 2. Results and Discussion

### 2.1. Effect of PAN/PVP Mass Ratio on Viscosity and Conductivity of Spinning Solutions

The viscosity and conductivity of the electrospinning solution have a significant effect on the morphology of NFMs. Under the condition of keeping the same content of SnCl_2_ in the spinning solution (with a mass ratio of 0.6:1 relative to PAN/PVP), PAN/PVP spinning solutions with different mass ratios were used for electrospinning, and their viscosity and conductivity were shown in Table 1. When the content of PVP increased, the solution viscosity decreased, which was due to the fact that PVP molecules were smaller and could reduce the intertwining of polymer chains. In addition, PVP was able to better solvate ions and promote ion migration [28]. Therefore, as the PVP content in the solution increased, the mobility of ions in the solution increased, leading to an increase in the solution conductivity.

### 2.2. Effect of PAN/PVP Mass Ratio on Morphology of Electrospun Nfms

**Figure 1:**
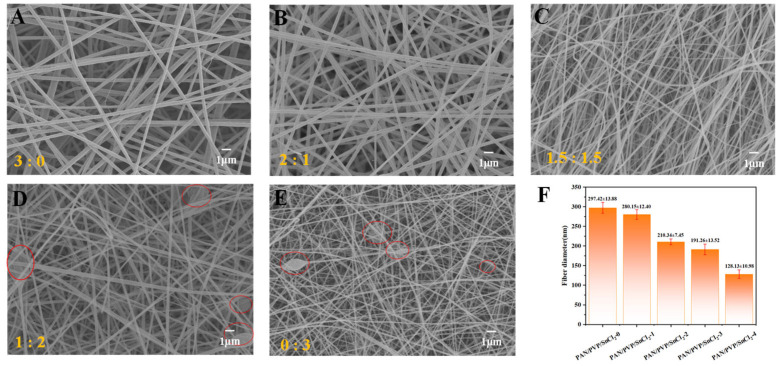
SEM images of (**A**) PAN/PVP/SnCl_2_-0; (**B**) PAN/PVP/SnCl_2_-1; (**C**) PAN/PVP/SnCl_2_-2; (**D**) PAN/PVP/SnCl_2_-3; and (**E**) PAN/PVP/SnCl_2_-4. (**F**) Average fiber diameters of the five NFMs.

Scanning electron microscopy (SEM) images of electrospun NFMs with different PAN/PVP mass ratios (PAN/PVP/SnCl_2_-X (X = 0, 1, 2, 3, 4) NFMs) were shown in Figure 1A–E. It could be seen that the fiber diameter of NFMs decreased with the increasing PVP content. This was due to the gradual decrease in the spinning solution viscosity and the increase in the solution conductivity, which made the jet stretch more fully during the spinning process [29]. However, when the PVP content was too much, the beaded structures formed due to the low viscosity of the spinning solution (Figure 1D,E). Therefore, PAN/PVP/SnCl_2_-2 NFM had the most uniform fiber diameter distribution, as shown in Figure 1F and Table S1. This indicated that the appropriate addition of PVP could improve the morphology and fiber diameter distribution of NFMs.

### 2.3. Morphological and Structural Analysis of Sn-C NFMs

**Figure 2:**
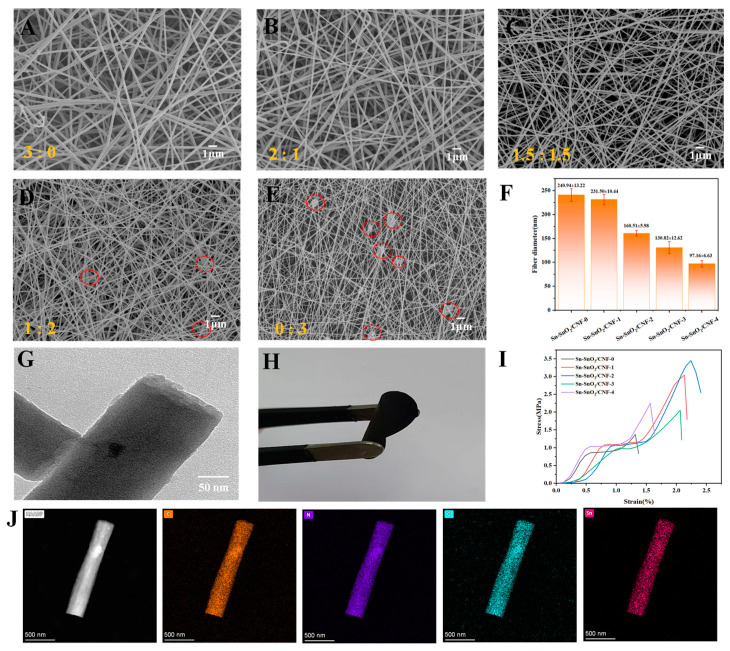
SEM images of (**A**) Sn-SnO_2_/CNF-0; (**B**) Sn-SnO_2_/CNF-1; (**C**) Sn-SnO_2_/CNF-2; (**D**) Sn-SnO_2_/CNF-3; and (**E**) Sn-SnO_2_/CNF-4. (**F**) Average fiber diameters of Sn-C NFMs. (**G**) Transmission electron microscope (TEM) images of the Sn-SnO_2_/CNF-2. (**H**) Binder-free electrode. (**I**) Tensile properties of five samples. (**J**) High-resolution TEM image of Sn-SnO_2_/CNF-2 and its element mapping of C, N, O, Sn.

Figure 2A–E presented the microscopic morphology of Sn-C NFMs (Sn-SnO_2_/CNF-X (X = 0, 1, 2, 3, 4)) after high-temperature carbonization at 700 °C. It could be seen that these Sn-C NFMs obtained from carbonization still maintained a three-dimensional network structure, and the trend of their fiber diameter changes was consistent with that of electrospun NFMs before carbonization, but the fiber diameter under corresponding ratios was finer than that before carbonization. As displayed in Figure 2F and Table S2, the average diameter of Sn-SnO_2_/CNF-2 obtained was 160.51 ± 5.98 nm, and its fiber diameter distribution was the most uniform due to the most uniform fiber diameter distribution of its precursor (PAN/PVP/SnCl_2_-2 NFM). While Sn-SnO_2_/CNF-3 and Sn-SnO_2_/CNF-4 had relatively poor diameter distribution uniformity and still kept the beaded fiber structures.

From Figure 2G, it could be seen that the Sn-based nanoparticles were well encapsulated in the nanofibers, and Sn-SnO_2_/CNF-2 could withstand a certain degree of bending, as shown in Figure 2H, exhibiting excellent flexibility. Figure S1 exhibited its short-range orderly amorphous characteristics [30]. Figure 2I displayed the effect of PVP addition on the mechanical properties of Sn-C NFMs. It could be seen that the mechanical properties of the Sn-C NFMs prepared by blending PVP with PAN were significantly improved due to the interaction of the strong polar groups between PVP and PAN, which led to an increase in the intermolecular force [27]. Moreover, as the PAN/PVP mass ratio increased, the mechanical properties of Sn-C NFMs first increased and then decreased. When the PAN/PVP mass ratio was 1.5:1.5, the mechanical properties of Sn-SnO_2_/CNF-2 were optimal, with a break strength of 3.45 ± 0.35 MPa and an elongation at break of 2.24% at this time. It might be because Sn-SnO_2_/CNF-2 had the optimal morphology with uniform fiber thickness, which enabled it to withstand greater tensile forces, thus providing a good foundation for its use as a self-supporting anode. With the further increase in PVP content, the mechanical properties of Sn-C NFMs showed a decreasing trend, which was related to their uneven diameter distribution of nanofibers and the emergence of beaded structures, resulting in the inability to evenly disperse the stress on the fibers during the stretching process. In addition, as shown in Figure 2J, it could be clearly seen that the elements of C, N, O, and Sn were uniformly distributed on Sn-SnO_2_/CNF-2, which was favorable for the storage and migration of lithium ions. Moreover, the atomic ratio of C and Sn was about 0.58 (Figure S2), which was equivalent to the addition ratio of Sn element in the spinning solution, indicating that Sn element was almost lost throughout the preparation process.

**Figure 3:**
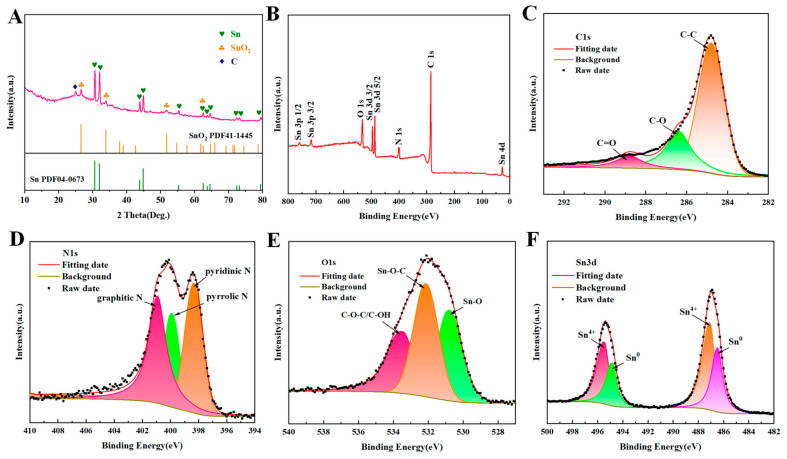
(**A**) XRD pattern of Sn-SnO_2_/CNF-2. (**B**) Full; (**C**) C 1s; (**D**) N 1s; (**E**) O 1s and (**F**) Sn 3d XPS spectra of Sn-SnO_2_/CNF-2.

To determine the crystal structure and crystallinity of Sn-SnO_2_/CNF-2, X-ray diffraction (XRD) analysis was performed, as shown in Figure 3A. From the XRD pattern of Sn-SnO_2_/CNF-2, it could be seen that the (002) crystal plane of carbon materials appeared around 2θ = 25°, indicating partial graphitization of carbon materials [31,32]. The diffraction peaks at 2θ = 30.6°, 32°, 43.9°, 44.9°, 55.3°, 62.5°, 63.8°, 64.6°, 72.4°, 73.2° and 79.5° corresponded to the (200), (101), (220), (211), (301), (112), (400), (321), (420), (411) and (312) crystal planes of the tetragonal Sn crystal (JCPDS: 04-0673) [33,34]. In addition, the diffraction peaks at 26.1°, 33.89° and 51.78° were consistent with the (110), (101) and (211) crystal planes of SnO_2_ (JCPDS:41-1445) [35]. The reason for the coexistence of SnO_2_ and Sn was that SnCl_2_ was first oxidized to SnO_2_ in the pre-oxidation, and then SnO_2_ was reduced to Sn in the high-temperature carbonization stage. However, due to the incomplete carbothermal reduction reaction of SnO_2_, SnO_2_ still partially existed.

To further understand the surface composition and chemical valence of Sn-SnO_2_/CNF-2, X-ray photoelectron spectroscopy (XPS) testing and analysis were performed. As shown in Figure 3B, the peaks of C, N, O and Sn elements appearing in the XPS full spectrum indicated their presence in Sn-SnO_2_/CNF-2, which was consistent with the results of element mapping. In the C1s fine spectrum (Figure 3C), three characteristic peaks located at 284.8 eV, 286.4 eV, and 288.8 eV corresponded to C-C, C-O, and C=O bonds [36], respectively. The N1s fine spectrum was fitted with three peaks (Figure 3D), which were pyridinic N (398.4 eV), pyrrolic N (399.9 eV), and graphite N (401.0 eV), indicating the presence of nitrogen doping in amorphous carbon [37,38]. Figure 3E showed the fine spectrum of O1s with three peaks observed at 530.8 eV, 532.1 eV and 533.5 eV, corresponding to Sn-O, Sn-O-C, and C-OH/C-O-C, respectively [39]. Professionally, the abundant Sn-O-C covalent bonds enhanced the interfacial interaction between SnO_x_ and carbon materials, which was beneficial for the tight anchoring of SnO_x_ in the carbon skeleton, forming a stable whole, reducing the intrinsic resistance of the composite and improving its conductivity. In the Sn 3d fine spectrum (Figure 3F), two humps located at 486.5 eV and 495.5 eV were exhibited, corresponding to Sn 3d 3/2 and Sn 3d 5/2, indicating that Sn had two valence states, Sn^0^ and Sn^4+^ [40]. This was consistent with the XRD analysis results, indicating that Sn elements existed in a multivalent state mixed in the material [41].

**Figure 4:**
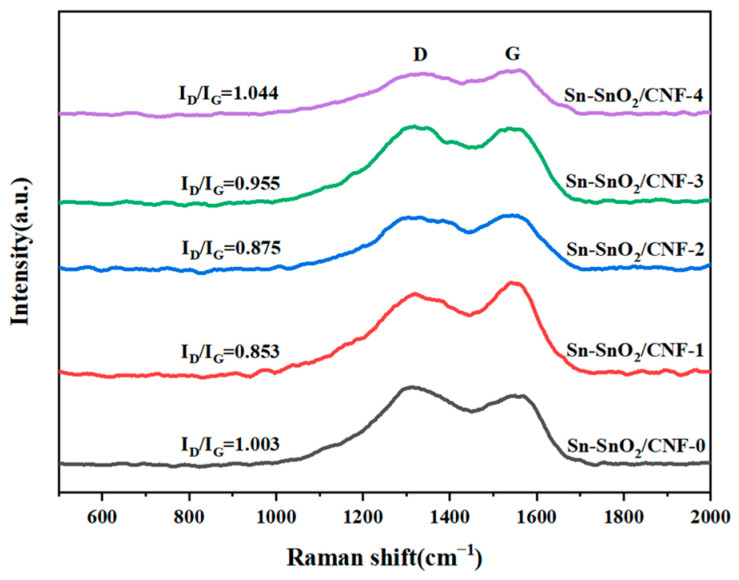
Raman spectra of the five samples.

In order to investigate the effect of PVP addition on the crystal structure of Sn-C NFMs, Raman spectroscopy test were conducted on them, as shown in Figure 4. The characteristic peak located near 1327 cm^−1^ was called D-band, which was mainly related to the lattice defects and the degree of disorder in the carbon material, and the other characteristic peak located near 1545 cm^−1^ was called G-band, which was related to the in-plane stretching vibration of sp^2^ hybridized carbon atoms [42,43]. The degree of graphitization of materials could be analyzed by calculating the intensity ratio (I_D_/I_G_) between the D peak of disordered structures and the G peak of graphitic structures. The smaller the I_D_/I_G_ ratio, the better the structural orderliness of the carbon material, indicating a higher degree of graphitization and conductivity of the material [44]. The corresponding I_D_/I_G_ values for Sn-SnO_2_/CNF-0, Sn-SnO_2_/CNF-1, Sn-SnO_2_/CNF-2, Sn-SnO_2_/CNF-3, Sn-SnO_2_/CNF-4 were 1.003, 0.853, 0.875, 0.955, 1.044, respectively. This indicated that the degree of graphitization of Sn-C NFMs first increased and then decreased with the increase in PVP content. Among them, Sn-SnO_2_/CNF-4 had the largest I_D_/I_G_ values, indicating the highest degree of residual carbon disorder and structural defects during pyrolysis. This was mainly related to the chain breaking and cross-linking reaction of pure PVP during heat treatment, making it difficult to form a stable graphite-like structure. Therefore, adding an appropriate amount of PVP to PAN could induce the formation of more graphitized crystal structures in the fabricated Sn-C NFMs, further enhancing the degree of graphitization and electrical conductivity of the materials. The electrical conductivity of electrodes were shown in Table S3, which further confirmed that a moderate addition of PVP could enhance the electrical conductivity of Sn-C NFMs.

### 2.4. Electrochemical Performance of Sn-C NFMs

**Figure 5:**
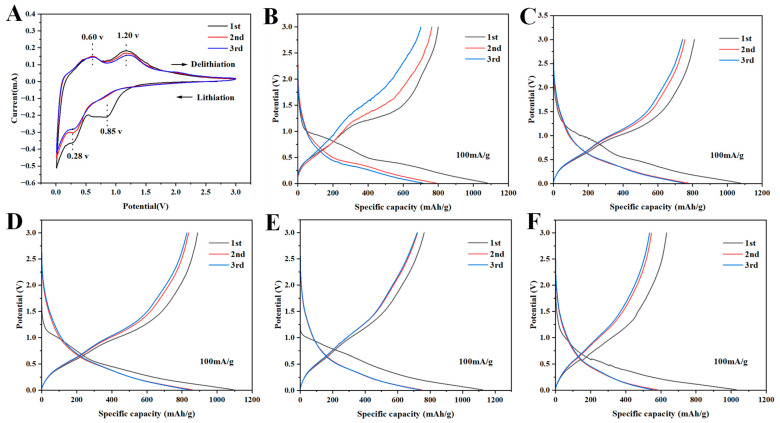
(**A**) CV curves of Sn-SnO_2_/CNF-2. (**B**) Galvanostatic charge–discharge (GCD) curves of Sn-SnO_2_/CNF-0; (**C**) Sn-SnO_2_/CNF-1; (**D**) Sn-SnO_2_/CNF-2; (**E**) Sn-SnO_2_/CNF-3; (**F**) Sn-SnO_2_/CNF-4.

Figure 5A exhibited the first three successive cyclic voltammetry (CV) curves of the Sn-SnO_2_/CNF-2 electrode at a scan rate of 0.1 mV/s. In the first cathodic scan, the reduction peaks appeared at around 0.85 V, corresponding to the generation of SEI films and the reversible reaction of SnO_2_ and Li^+^ (SnO_2_ + 4Li^+^ + 4e^−^ → Sn + 2Li_2_O) [45,46]. The disappearance of reduction peak in the subsequent cycle indicated the existence of an irreversible process in this reaction. The reduction peak at around 0.28 V corresponded to the embedding of Li^+^ in carbon and the alloying process of Sn with lithium (Sn + xLi^+^ + xe^−^ ↔ Li_x_Sn), which was reversible and still observed in the following cycles [45,47]. During the subsequent anodization scans, two oxidation peaks appeared around 0.6 V and 1.2 V, representing the delithiation reaction of the Li_x_Sn alloy and the reversible reaction of Li_2_O with Sn to produce SnO_2_ and lithium, respectively (Li_x_Sn → Sn → SnO_2_) [48,49,50]. Almost overlapping oxidation curves could be seen in the two subsequent cycles, suggesting that the Sn-SnO_2_/CNF-2 electrode had high electrochemical reversibility and good cycling stability.

Figure 5B–F showed the GCD curves of Sn-C NFMs for the first three cycles at a current density of 100 mA/g. The first charge/discharge specific capacities of Sn-SnO_2_/CNF-0, Sn-SnO_2_/CNF-1, Sn-SnO_2_/CNF-2, Sn-SnO_2_/CNF-3, and Sn-SnO_2_/CNF-4 electrodes were 800.17/1079.96 mAh/g, 810.45/1076.67 mAh/g, 888.84/1093.11 mAh/g, 762.85/1124.50 mAh/g, and 632.58/1029.72 mAh/g, respectively, and their initial Coulombic efficiencies (ICEs) were calculated to be 74.1%, 75.3%, 81.3%, 67.8%, and 61.4%, respectively. Sn-SnO_2_/CNF-2 had the highest ICE value due to its suitable nitrogen content and optimal mechanical properties derived from the appropriate addition of PVP.

**Figure 6:**
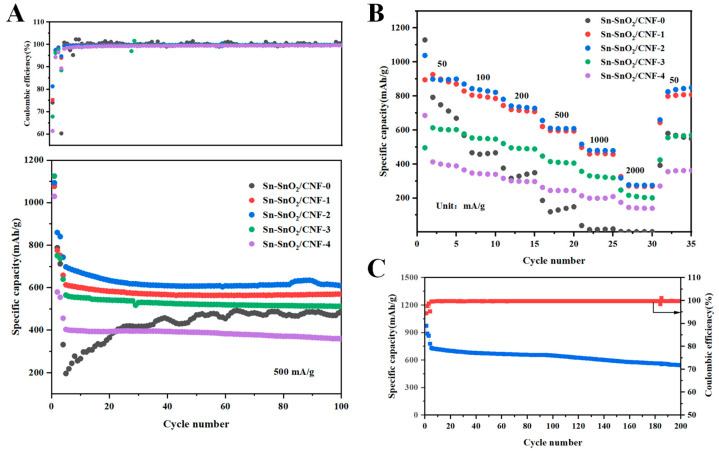
(**A**) Cycling performance and (**B**) rate capabilities of five samples. (**C**) Long-term cycling performance of Sn-SnO_2_/CNF-2 electrode at 500 mA/g.

Figure 6A compared the performance of five samples at a current density of 500 mA/g after 100 cycles. Among them, the first three cycles showed the activation of the half-cells assembled from the samples at 100 mA/g, allowing the active species to fully participate in the reaction. In the first three cycles, all five samples initially exhibited high discharge specific capacity and rapid decay of specific capacity, which could be attributed to the irreversible capacity loss of the Sn-C NFMs during the pre-alloying reaction. Whereas, the subsequent cycles were carried out at 500 mA/g, and the Coulombic efficiencies of samples all rapidly increased to about 99.7% in the subsequent cycles. The Sn-SnO_2_/CNF-2 electrode consistently showed the most remarkable stability, maintaining a high discharge specific capacity of 607.28 mAh/g even after 100 cycles, while the Sn-SnO_2_/CNF-1, Sn-SnO_2_/CNF-3, and Sn-SnO_2_/CNF-4 electrodes only remained specific capacities of 570.39 mAh/g, 512.15 mAh/g, and 360.29 mAh/g, respectively, after 100 cycles under the same conditions. These results indicated that the moderate addition of PVP could improve the stability of Sn-C nanofiber structures, thus enhancing the cycling performance of the electrodes.

Figure 6B showed the rate performance of five samples at different current densities. The discharge specific capacities of Sn-SnO_2_/CNF-2 electrode at 50, 100, 200, 500, 1000, and 2000 mA/g were 899.54, 821.77, 727.89, 608.39, 478.80, and 275.01 mAh/g, respectively. When the current density returned to 50 mA/g, the specific capacity of Sn-SnO_2_/CNF-2 electrode recovered to 848.43 mAh/g, showing its good rate performance. On the contrast, Sn-SnO_2_/CNF-0 electrode had poor rate performance, especially with extremely low discharge specific capacity at a high current density. Furthermore, Figure 6C illustrated that the Sn-SnO_2_/CNF-2 electrode still maintained a specific capacity of 543.78 mAh/g after 200 cycles at 500 mA/g, which further proved its good long-term cycling stability. Table S4 summarized the electrochemical performances of reported Sn-based carbon anodes for high-performance LIBs [51,52,53,54,55]. It could be seen that Sn-SnO_2_/CNF-2 exhibited outstanding electrochemical performances, particularly in terms of ICE and cycling stability.

**Figure 7:**
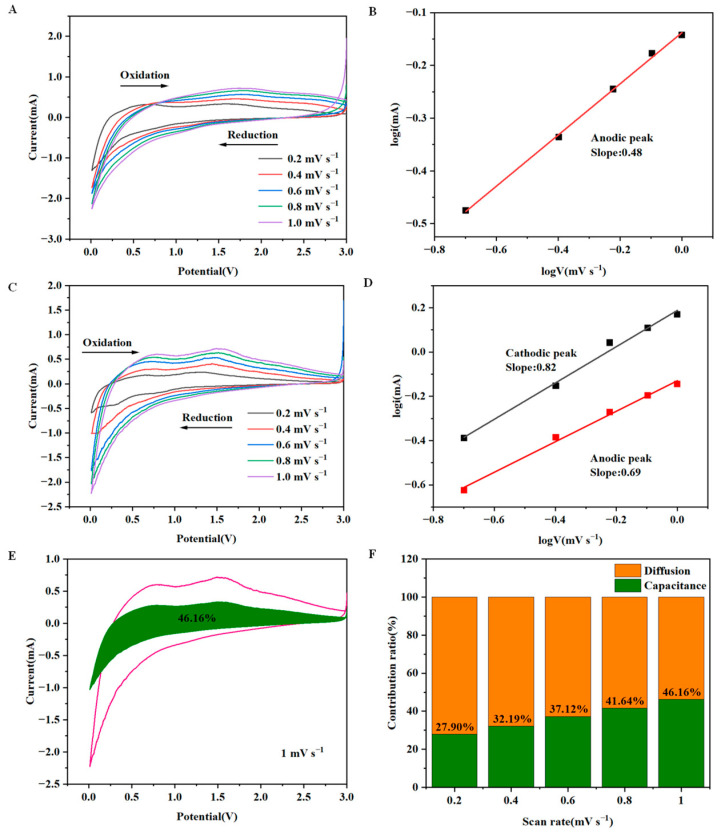
(**A**) CV curves of Sn-SnO_2_/CNF-0 electrode at different scan rates and (**B**) logarithmic relationship between peak current and scan rate. (**C**) CV curves of Sn-SnO_2_/CNF-2 electrode at different scan rates and (**D**) logarithmic relationship between peak current and scan rate. (**E**) Capacitance and diffusion-controlled contributions of Sn-SnO_2_/CNF-2 electrode at 1 mV s^−1^. (**F**) Capacitance and diffusion-controlled contributions of Sn-SnO_2_/CNF-2 electrode at different scan rates.

For illustrating the lithium storage mechanism of Sn-C NFM electrodes, the CV curves of Sn-SnO_2_/CNF-0 and Sn-SnO_2_/CNF-2 electrodes were tested at scanning rates of 0.2, 0.4, 0.6, 0.8 and 1.0 mV/s, respectively. It could be seen from Figure 7A that the cathodic peaks of Sn-SnO_2_/CNF-0 electrode at different scan rates were not obvious. Therefore, only the b value of anodic peaks were obtained from Equation (S1), which was about 0.5 (Figure 7B), indicating that the storage kinetics of Li^+^ was diffusion controlled, further explaining the poor rate performance of Sn-SnO_2_/CNF-0 electrode. However, as shown in Figure 7C, the Sn-SnO_2_/CNF-2 electrode exhibited similar shapes at different scan rates, indicating its remarkable reversibility and stability. The b values of the cathodic and anodic peaks obtained from Equation (S1) were 0.82 and 0.69, respectively, as shown in Figure 7D, indicating that the storage kinetics of Li^+^ were a combination of diffusion control and capacitance. According to Equations (S1) and (S2), the capacitance contribution percentage of Sn-SnO_2_/CNF-2 electrode at 1 mV s^−1^ was calculated to be 46.16%, as displayed in Figure 7E. Figure 7F exhibited the capacitance and diffusion contribution percentages of Sn-SnO_2_/CNF-2 electrode at different scan rates, illustrated that its capacitive contribution increased with the increase in scan rate.

**Figure 8:**
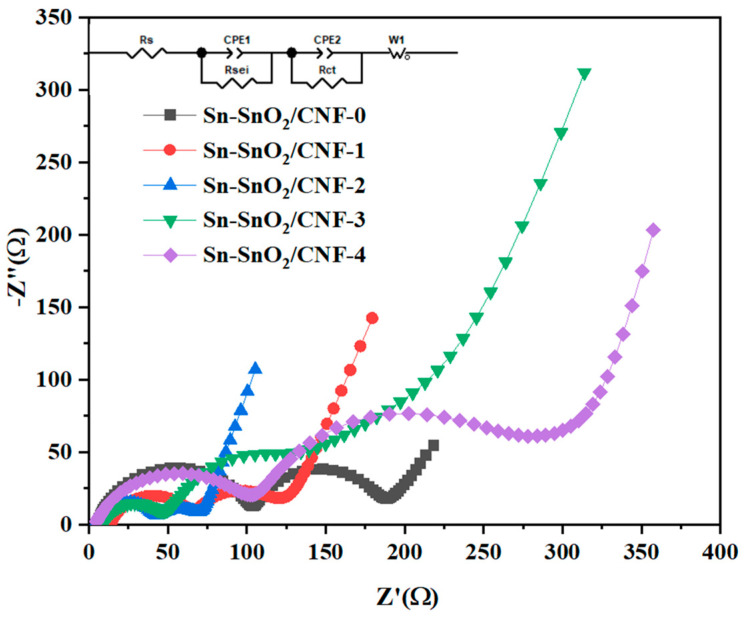
EIS plots of five samples.

Electrochemical impedance spectroscopy (EIS) measurements were performed on the five electrodes separately, and the obtained Nyquist plots and the specific data fitted by the equivalent circuit were shown in Figure 8 and Table S5. In the high-frequency field, each plot presented two semicircles. The first semicircle denoted the resistance of diffusive migration of Li^+^ through the SEI films, corresponding to R_SEI_ in the equivalent circuit. The second semicircle represented the charge transfer resistance, which was R_ct_ in the equivalent circuit. The intersection with the real axis referred to the electrolyte resistance, corresponding to R_S_ in the circuit. In low-frequency fields, the slope of the straight line reflected the diffusion capacity of the ions [56,57]. The R_ct_ values of Sn-SnO_2_/CNF-0, Sn-SnO_2_/CNF-1, Sn-SnO_2_/CNF-2, Sn-SnO_2_/CNF-3, and Sn-SnO_2_/CNF-4 electrodes were 78.24 Ω, 40.36 Ω, 29.5 Ω, 40.74 Ω, and 132.2 Ω, respectively, and their R_SEI_ values were 99.35 Ω, 50.63 Ω, 37.1 Ω, 31.72 Ω, 97.89 Ω, respectively, indicating that the R_ct_ of Sn-SnO_2_/CNF-2 was obviously smallest and its SEI films formed were also more uniform and stable. In addition, EIS measurements were performed on Sn-SnO_2_/CNF-0 and Sn-SnO_2_/CNF-2 electrodes after 100 cycles at 500 mA/g to investigate their internal changes before and after cycling, and their corresponding Nyquist plots and specific fitting data were shown in Figure S3 and Table S6. The results showed that after 100 cycles, the R_ct_ and R_SEI_ values of Sn-SnO_2_/CNF-0 and Sn-SnO_2_/CNF-2 all increased. It was worth noting that the R_SEI_ value of Sn-SnO_2_/CNF-0 significantly increased, while the R_SEI_ value of Sn-SnO_2_/CNF-2 slightly increased, further indicating that the SEI film formed by Sn-SnO_2_/CNF-2 remained uniform and stable even after multiple cycles.

**Figure 9:**
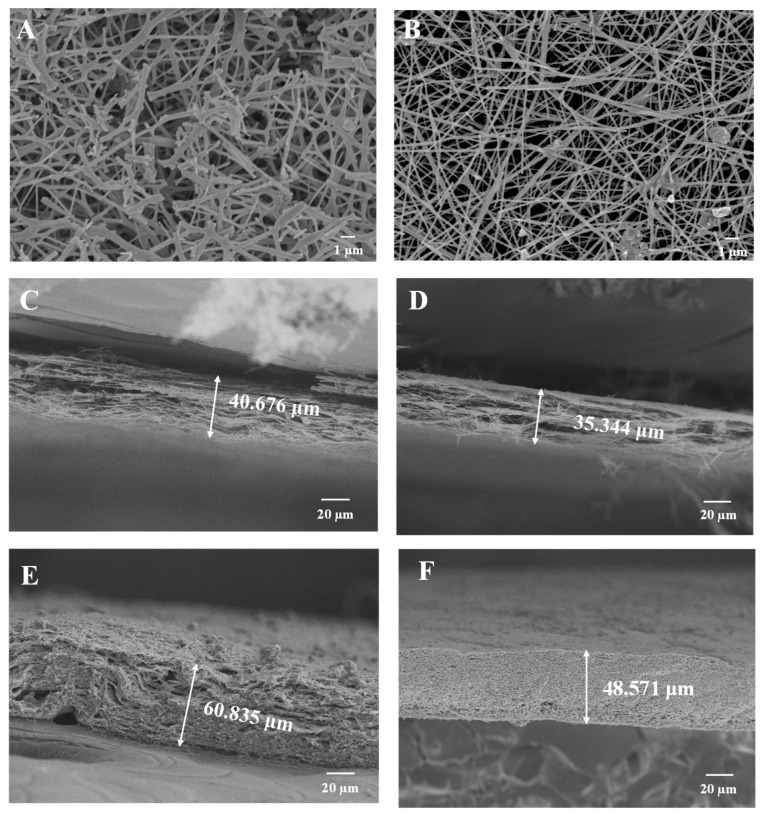
(**A**) Top-view SEM images of Sn-SnO_2_/CNF-0 and (**B**) Sn-SnO_2_/CNF-2 after 100 cycles. (**C**) Cross-sectional SEM images of Sn-SnO_2_/CNF-0 and (**D**) Sn-SnO_2_/CNF-2 before cycling. (**E**) Cross-sectional SEM images of Sn-SnO_2_/CNF-0 and (**F**) Sn-SnO_2_/CNF-2 after 100 cycles.

Figure 9 demonstrated the morphology of samples in top view and cross-section. It could be clearly seen that after 100 cycles, the nanofibers in Sn-SnO_2_/CNF-0 electrode were severely broken (Figure 9A), whereas Sn-SnO_2_/CNF-2 electrode basically maintained its original fiber morphology with less damage (Figure 9B). The cross-sectional SEM images of Sn-SnO_2_/CNF-0 electrode before (Figure 9C) and after (Figure 9E) cycling indicated a sharp increase in electrode thickness from 40.676 μm to 60.835 μm, with a volume expansion rate of 49.56% calculated by Equation (S3). As shown in Figure 9D,F, the thickness of Sn-SnO_2_/CNF-2 electrode increased from 35.344 μm to 48.571 μm, with a volume expansion rate of 37.42%, which further indicated that the Sn-SnO_2_/CNF-2 electrode containing an appropriate amount of PVP had better cycle stability due to its best mechanical property and structural stability.

## 3. Materials and Methods

### 3.1. Materials

PAN powders (M_w_ = 150,000, AR, 98%) were supplied by Beijing Bailing Branch (Beijing, China). PVP powders (M_w_ = 1,300,000, AR, 99%) and SnCl_2_·2H_2_O (AR, 98%) were supplied by Shanghai Aladdin Biochemical Technology Co, Ltd. (Shanghai, China). N,N-Dimethylformamide (DMF) was purchased from Sinopharm Chemical Reagent Co, Ltd. (Shanghai, China).

### 3.2. Preparation of Sn-C NFM Self-Supporting Anode

**Figure 10:**
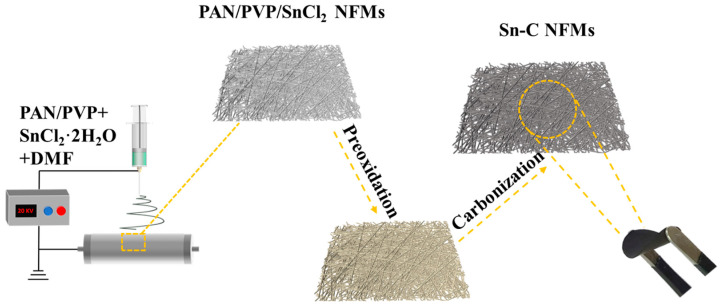
Flowchart for the preparation of Sn-C NFM self-supporting anodes.

PAN/PVP/SnCl_2_ NFMs were prepared by single-needle electrospinning. First, 10 mL spinning solutions with a total solute mass fraction of 10 wt% were prepared using DMF as the solvent as well as PAN and PVP as solutes (PAN to PVP mass ratios of 3:0, 2:1, 1.5:1.5, 1:2, and 0:3, respectively). Then, SnCl_2_⋅2H_2_O (with a mass ratio of 0.6:1 to the total solute) was added to the prepared solutions. After stirring at room temperature for 12 h, PAN/PVP/SnCl_2_ spinning solutions with different PAN/PVP mass ratios were obtained. The spinning voltage was 20 kV, the flow rate was 0.5 mL/h, the receiving distance was 18 cm, and the rotating speed of the drum receiver was 150 r/min. The environmental temperature and relative humidity were 25 °C and 50%, respectively. The electrospun PAN/PVP/SnCl_2_ NFMs with different PAN/PVP mass ratios were noted as PAN/PVP/SnCl_2_-0 (3:0), PAN/PVP/SnCl_2_-1 (2:1), PAN/PVP/SnCl_2_-2 (1.5:1.5), PAN/PVP/SnCl_2_-3 (1:2), PAN/PVP/SnCl_2_-4 (0:3), respectively. Finally, the electrospun PAN/PVP/SnCl_2_ NFMs were pre-oxidized by heating to 250 °C at a rate of 5 °C/min in air atmosphere and holding for 2 h, and then carbonized at 700 °C for 2 h in nitrogen atmosphere to obtain Sn-C NFM self-supporting anode with a mass of about 1.5 mg, labeled as Sn-SnO_2_/CNF-X (X = 0, 1, 2, 3, 4), respectively. The whole preparation process of Sn-C NFM self-supporting anodes was shown in Figure 10.

## 4. Conclusions

In this paper, self-supporting flexible Sn-C NFM electrodes were successfully prepared as high-performance LIB anodes, in which Sn-based nanoparticles with high theoretical specific capacity were uniformly embedded into the carbon nanofibers through electrospinning and annealing. The synergistic effect between Sn-based nanoparticles and carbon nanofibers effectively suppressed the volume expansion of Sn-based nanoparticles during charging and discharging, resulting in high specific capacity and good cycling performance of the electrode. In addition, by adjusting the PAN/PVP mass ratio in the spinning solutions, Sn-C NFMs with excellent mechanical and electrochemical properties were obtained, and the optimal PAN/PVP mass ratio was determined to be 1.5:1.5, illustrating that the moderate addition of PVP could prepare the optimal Sn-C NFM electrode with the best morphology, the most uniform fiber diameter distribution, and the best mechanical properties, thereby making it have good flexibility, well structural stability, and excellent electrochemical performances. Accordingly, the obtained optimal self-supporting Sn-SnO_2_/CNF-2 anode had a discharge specific capacity of 607.28 mAh/g after 100 cycles at a current density of 500 mA/g. It was remarkable that even after 200 cycles, the Sn-SnO_2_/CNF-2 anode still maintained a specific capacity of 543.78 mAh/g and well retained its original fiber structure, proving its good long-term cycling stability. This demonstrated that the self-supporting Sn-SnO_2_/CNF-2 anode had great potential for advanced energy storage.

## Figures and Tables

**Table 1:** Viscosity and conductivity of solutions with different PAN/PVP ratios.

Label	PAN/PVP	Viscosity (mPa·s)	Conductivity (mS/cm)
PAN/PVP/SnCl_2_-0	3:0	487 ± 17	10.436 ± 0.048
PAN/PVP/SnCl_2_-1	2:1	444 ± 11	10.888 ± 0.017
PAN/PVP/SnCl_2_-2	1.5:1.5	287 ± 4	11.824 ± 0.015
PAN/PVP/SnCl_2_-3	1:2	251 ± 9	12.474 ± 0.017
PAN/PVP/SnCl_2_-4	0:3	246 ± 6	12.664 ± 0.042

## Data Availability

The original contributions presented in the study are included in the article/Supplementary Material, further inquiries can be directed to the corresponding author.

## Supplementary Materials

The following supporting information can be downloaded at https://www.mdpi.com/article/10.3390/molecules30081740/s1: Figure S1: High-resolution TEM image of Sn-SnO_2_/CNF-2; Figure S2: High-resolution SEM image of Sn-SnO_2_/CNF-2 and element mapping of C, N, O, Sn; Figure S3: EIS of Sn//SnO_2_/CNF-0 and Sn/SnO_2_/CNF-2 after 100 cycles; Table S1: Fiber diameters of PAN/PVP/SnCl_2_-X NFMs; Table S2: Fiber diameters of Sn-C NFMs; Table S3: Electrical conductivity of electrodes; Table S4: Comparative analysis of Sn-based carbon materials anode for high-performance lithium-ion batteries; Table S5: Summary of EIS fitting results of Sn-C NFM electrodes before cycling; Table S6: Summary of EIS fitting results of Sn/SnO_2_/CNF-0 and Sn/SnO_2_/CNF-2 electrodes after cycling.
