# Supplementary material for: Self-Supporting Sn-Based Carbon Nanofiber Anodes for High-Performance Lithium-Ion Batteries
Source: Molecules. 2025 Apr 13;30(8):1740. doi: 10.3390/molecules30081740 (PMC12029184; doi:10.3390/molecules30081740)
Supplement: Supplementary file 1 [file molecules-30-01740-s001.zip › molecules-3556749-supplementary.pdf]

Supporting Information

Self-Supporting Sn-Based Carbon Nanofiber Anodes for High-Performance Lithium-Ion Batteries

Jingjie Xie[1] and Lan Xu[1,2,*]

[1]National Engineering Laboratory for Modern Silk, College of Textile and Clothing Engineering, Soochow University, Suzhou 215123, China; xiejingjie2023@163.com

[2]Jiangsu Engineering Research Center of Textile Dyeing and Printing for Energy Conservation, Discharge Reduction and Cleaner Production (ERC), Soochow University, Suzhou 215123, China.

*Correspondence: lanxu@suda.edu.cn

Material characterization

The morphology and structure of samples were characterized by field emission scanning electron microscopy (FE-SEM, S4800), high-resolution field emission scanning electron microscopy (HR-SEM, Regulus 8230), transmission electron microscope (TEM, HT7700) and high-resolution transmission electron microscope (HR-TEM, Talos F200XTa). The crystal structures of samples were analyzed by X-ray diffraction (XRD, D8 Advance, CuKα, scanning speed 2° min^{-1}, 2Theta = 10°-80°) and Raman spectroscopy (Raman, Xplora Pius, 532 nm laser). X-ray photoelectron spectroscopy (XPS, Thermo Scientific KAlpha) was used to measure the elemental compositions and chemical structures of the composites of samples. The tensile properties of Sn-carbon nanofiber membranes (Sn-C NFMs) were tested using a universal material tester (Instron 3365, USA) according to GB/T 3923.1-1997, with a sample size of 10 mm × 40 mm, a gauge length of 20 mm, a preload force of 0.2 cN, a tensile speed of 10 mm/min, and thickness measured by a thickness tester (BK-5183, China).

Electrochemical measurement and characterization

The electrochemical properties of the prepared electrode were measured using an assembled 2025-type coin cell, with the prepared electrode serving as the working electrode, lithium metal foil was used as the counter electrode, and microporous polyethylene films (Celgard 2400) as the separator. The prepared self-supporting Sn-C NFMs were cut into circular shapes with a diameter of 12 mm and directly used as the working electrode, in which the mass of each electrode was approximately 1.5 mg. The electrolyte was prepared by dissolving 1.0 M $LiPF_6$ in ethylene carbonate (EC): dimethyl carbonate (DMC): ethyl methyl carbonate (EMC) = 1:1:1 vol%. The cell was assembled in a glove box filled with high purity argon (O_2 and H_2O < 0.01 ppm). The cyclic voltammetry (CV) and electrochemical impedance spectroscopy (EIS) were carried out using CHI660E electrochemical workstation (Shanghai Chenhua Instrument,

China). Galvanostatic charge–discharge (GCD) measurements were carried out in the voltage range of 0.01-3.00 V. The rate capability and cyclic stability were tested by a battery test system (BTS, Neware Electronic Co. China) at 25 degrees Celsius. All cells underwent a 24-hour shelving process before testing. The electrical conductivity of Sn-C NFMs was measured using an ST-2258C four-point probe system (a multifunctional digital tester).

The relationship equations between the tested peak current (i) and scan rate (v) are as follows:

$$i_p = av^b \quad \text{(S1)}$$

$$i = k_1 v + k_2 v^{1/2} \quad \text{(S2)}$$

where b is a constant that can be obtained from the slope of the linear fit in the log(v) vs. log(i) plot. Typically, b = 1 indicates a capacitive process, b = 0.5 suggests diffusion control, and values between 0.5 and 1 imply a mixed diffusion capacitance behavior.

The volume expansion rate of electrode materials before and after cycling is determined from thickness changes, calculated as:

$$Expansion\ rate = \frac{\Delta h}{h_0} \times 100\% \quad \text{(S3)}$$

where Δh is the amount of change in thickness (i.e., the final thickness (h_f) minus the initial thickness (h_0)).

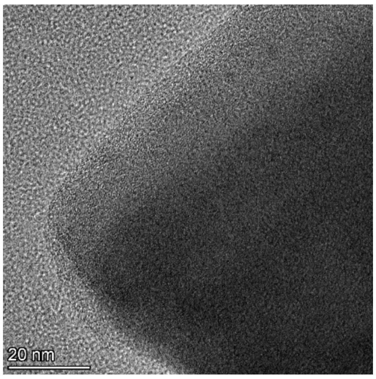

Figure S1. High-resolution TEM image of Sn-SnO_2/CNF-2.

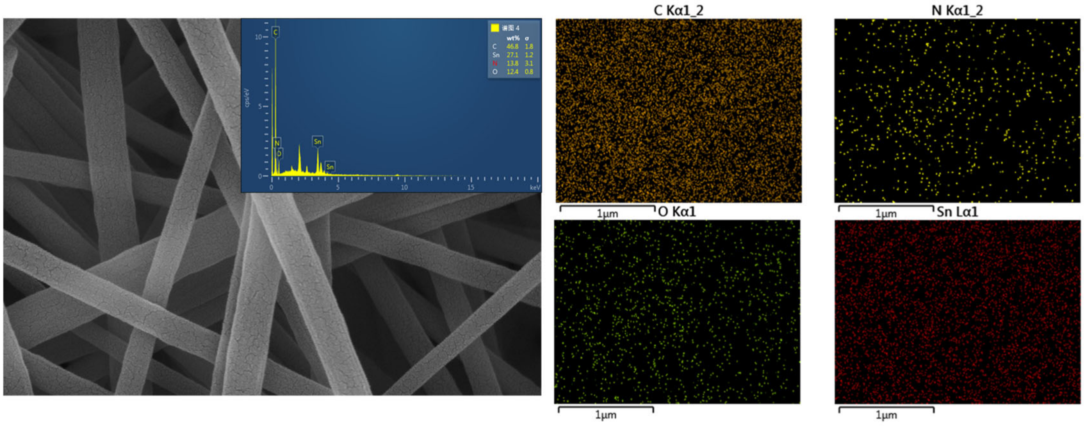

Figure S2. High-resolution SEM image of Sn-SnO_2/CNF-2 and element mapping of C, N, O, Sn.

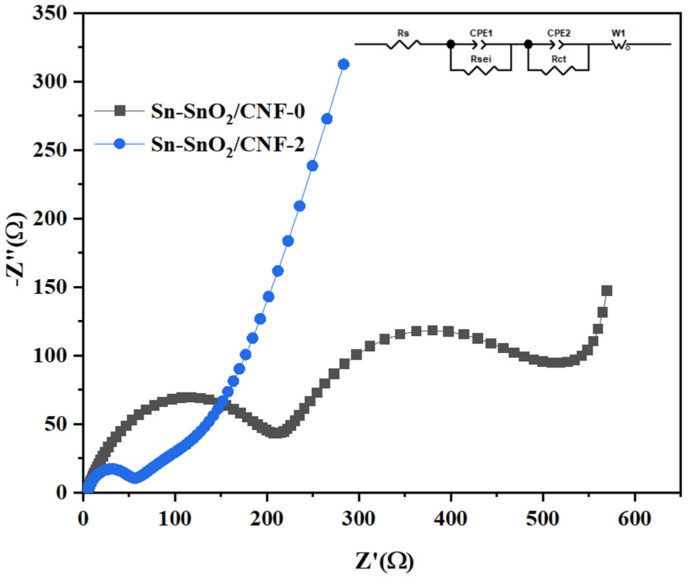

Figure S3. EIS of Sn/SnO_2/CNF-0 and Sn/SnO_2/CNF-2 after 100 cycles.

Table S1. Fiber diameters of PAN/PVP/$SnCl_2$-X NFMs.

Anode materials	PAN/PVP/ $SnCl_2$-0	PAN/PVP/ $SnCl_2$-1	PAN/PVP/Sn Cl_2-2	PAN/PVP/ $SnCl_2$-3	PAN/PVP/ $SnCl_2$-4
Average diameter (nm)	297.42	280.15	210.34	191.26	128.13
Standard deviation (nm)	50.09	44.72	26.87	48.78	39.62
Confidence interval (nm)	13.88	12.40	7.45	13.52	10.98

Table S2. Fiber diameters of Sn-C NFMs.

Anode materials	Sn-SnO_2/ CNF-0	Sn-SnO_2/ CNF-1	Sn-SnO_2/ CNF-2	Sn-SnO_2/ CNF-3	Sn-SnO_2/ CNF-4
Average fiber diameters (nm)	240.94	231.50	160.51	130.82	97.16
Standard deviation (nm)	47.68	37.67	21.56	45.52	23.92
Confidence interval (nm)	13.22	10.44	5.98	12.62	6.63

Table S3. Electrical conductivity of electrodes.

Sample	Sn-SnO_2/CNF-0	Sn-SnO_2/CNF-1	Sn-SnO_2/CNF-2	Sn-SnO_2/CNF-3
Electrical conductivity (S/m)	0.799	1.154	1.539	0.662

Table S4. Comparative analysis of Sn-based carbon materials anode for high-performance lithium-ion batteries.

Sample	Current density (mA/g)	ICE (%)	Cycles Speciffc capcity (mAh/g) /Current density / Cycles	Ref.
**P-Sn/C-N**	500	61.2	712.1/500/500	[51]
**Sn/NPCFs-0.5**	500	62.9	400/100/500	[52]
**SnSb-CNTs@NCNFs**	100	75	815/100/100	[53]
**Sn/SnO_2/AMCMB**	100	35	451/100/50	[54]
**Sn/g-C_3N_4**	100	33	~200/1000/100/	[55]
**Sn/SnO_2/CNF-2**	100	81.3	607.28/500/100	This work

Table S5. Summary of EIS fitting results of Sn-C NFM electrodes before cycling.

Anode materials	Sn-SnO_2/CNF-0	Sn-SnO_2/CNF-1	Sn-SnO_2/CNF-2	Sn-SnO_2/CNF-3	Sn-SnO_2/CNF-4
**R_{ct} (Ω)**	78.24	40.36	29.5	40.74	132.2
**R_{SEI} (Ω)**	99.35	50.63	37.1	31.72	97.89
**R_S (Ω)**	5.58	13.73	4.50	9.36	3.95
**Slope**	1.29	2.39	2.91	2.32	2.61

Table S6. Summary of EIS fitting results of Sn/SnO_2/CNF-0 and Sn/SnO_2/CNF-2 electrodes after cycling.

After 100 cycle	Sn-SnO_2/CNF-0	Sn-SnO_2/CNF-2
**R_{ct} (Ω)**	245.3	115.7
**R_{SEI} (Ω)**	210.2	44.81
**R_S (Ω)**	4.37	3.86
**Slope**	1.39	1.84
